# Effectiveness and Tolerability of Intranasal Esketamine in Treatment-Resistant Depression: Report of Two Clinical Cases

**DOI:** 10.1192/j.eurpsy.2023.1745

**Published:** 2023-07-19

**Authors:** A. Mercado-Rodríguez, C. Martín Requena, A. Cano Baena, I. Zorrilla Martínez, A. González-Pinto Arrillaga, L. Mar-Barrutia

**Affiliations:** 1 Osakidetza Basque Health Service, Araba University Hospital, Psychiatry Department; 2 Bioaraba, Mental Health and Childhood Research Group; 3 Department of Neurosciences, University of the Basque Country UPV/EHU; 4 CIBER of Mental Health (CIBERSAM), Institute of Health Carlos III; 5 Bioaraba, Mental Health and Childhood Research Group, Vitoria-Gasteiz, Spain

## Abstract

**Introduction:**

Major depressive disorder (MDD) is a mental health disorder characterised by persistently low mood; anhedonia; feelings of worthlessness and guilt; altered appetite, weight and sleep and suicidal ideation. About one-third of patients do not respond to available antidepressants (AD). Treatment-resistant depression (TRD) is a clinical term used to define a lack of response to two or more AD in patients with MDD that do not respond to other lines of treatment either. TRD is associated with an increased risk of relapse, hospitalisation and suicide. Esketamine is a non-competitive NMDAR antagonist that acts as an antidepressant by modulating glutamatergic neurotransmission, disturbed in MDD patients. It has recently been approved by the European Commission as a fast-acting nasal spray therapy for depression and suicidal ideation after showing effectiveness in TRD patients (Papakostas *et al.* JCP 2020; 81 4).

**Objectives:**

The aim of this study is to determine the effectiveness, safety and tolerability of intranasal esketamine in two TRD-diagnosed patients and to assess their clinical evolution.

**Methods:**

A prospective study was conducted describing the evolution of two TRD patients treated with intranasal esketamine. We used The Hamilton Depression Rating Scale (HDRS) to quantify the severity of their symptoms and assess their recovery over time, analyzing the score change from baseline to endpoint as a primary outcome of the study. We also applied the Addensbrooke Cognitive Examination (ACE-III) as a tool to establish their cognitive condition before therapy and its evolution. Changes in dosage during treatment, adverse effects, time required for onset of action, clinical outcomes and other variables were also measured.

**Results:**

Intranasal esketamine was administered twice a week during the first 4-week induction phase and weekly during the following 6-month maintenance phase. Dosage of antidepressant was determined depending on each patient’s age and clinical evolution, being 56 mg the initial dose for case 1 (57 years old) and 28 mg for case 2 (71 years old). This antidepressant was effective in both patients in a fast-acting way, with the onset of action occuring within the first two weeks. During the course of treatment, the HDRS score significantly decreased, associated with improvement and remission of depressive symptoms. Cognitive performance got better in both cases. None of the patients discontinued treatment due to adverse effects or lack of efficacy.

**Conclusions:**

Our data suggest that intranasal esketamine therapy is a good alternative in TRD patients, being effective, fast-acting and well-tolerated, with a manageable safety profile. Clinical stability was also observed in the medium-term follow-up after the end of treatment. This presents esketamine as a promising therapeutic and effective strategy in MDD patients who are either treatment-resistant or acutely suicidal.

**Disclosure of Interest:**

None Declared

